# The logarithmic relaxation process and the critical temperature of liquids in nano-confined states

**DOI:** 10.1038/srep33374

**Published:** 2016-09-27

**Authors:** Changjiu Chen, Kaikin Wong, Richard A. Mole, Dehong Yu, Suresh M. Chathoth

**Affiliations:** 1 Department of Physics and Materials Science, City University of Hong Kong, Hong Kong, 999077, P. R. China; 2 City University of Hong Kong Shenzhen Research Institute, Shenzhen, 518057, P. R. China; 3 Australian Nuclear Science and Technology Organization, Lucas Heights, NSW 2234, Australia

## Abstract

The logarithmic relaxation process is the slowest of all relaxation processes and is exhibited by only a few molecular liquids and proteins. Bulk salol, which is a glass-forming liquid, is known to exhibit logarithmic decay of intermediate scattering function for the β-relaxation process. In this article, we report the influence of nanoscale confinements on the logarithmic relaxation process and changes in the microscopic glass-transition temperature of salol in the carbon and silica nanopores. The generalized vibrational density-of-states of the confined salol indicates that the interaction of salol with ordered nanoporous carbon is hydrophilic in nature whereas the interaction with silica surfaces is more hydrophobic. The mode-coupling theory critical temperature derived from the QENS data shows that the dynamic transition occurs at much lower temperature in the carbon pores than in silica pores. The results of this study indicate that, under nano-confinements, liquids that display logarithmic β-relaxation phenomenon undergo a unique glass transition process.

Fluids confined in nanoscale interfaces are known to exhibit anomalous behaviour^1^,^2^,^3^,^4^,^5^,^6^,^7^,^8^. For example, water can be supercooled much below its homogeneous nucleation temperature (235 K at 1 atm) in nanopores^9^,^10^,^11^,^12^. Although, water freezes at much lower temperature in nanopores, the pore size and liquid pore-surface interaction are important in determining the degree of undercooling achieved^9^,^12^,^13^. For example, water in hydrophobic pores can be supercooled to much lower temperature than in hydrophilic pores^13^,^14^. Moreover, water confined in hydrophobic nanopores has been found to be progressively transformed to amorphous ice and the calorimetric glass-transition temperature of water decreases as low as 160 K in hydrophobic pores with diameters of less than 18 Å^11^,^13^. Not only water but also monoatomic liquids and several organic liquids have been found to show similar phenomena^15^,^16^. The above mentioned anomalous behaviour of fluids in nanoscale interfaces accompanied by changes in the atomic/molecular relaxation dynamics of fluids. Apart from the rotational relaxation process, molecules in a liquid undergo two types of relaxation processes. The β-relaxation, in which the molecules rattle in the cage formed by its neighbouring molecules and the α-relaxation, which is the long range molecular transport process. Both α and β-relaxation processes can be determined by measuring the intermediate scattering function (ISF)^17^. In general ISF, representing the β-relaxation process, decays exponentially with time. However, in recent years, an unusual decay of the fast β-relaxation process has been observed in certain liquids, in these cases the intermediate scattering function decays logarithmically with time^18^,^19^,^20^,^21^. Interestingly, the logarithmic relaxation process is the slowest of all relaxation processes and least observed in liquids.

Mode-coupling theory (MCT), in its idealized version, has predicted the logarithmic β-relaxation process close to the higher-order glass transition singularities^22^. The theory is based on a set of coupled nonlinear equations of motion for the autocorrelation function; the coupling coefficients in these equations are a function of structural parameters such as the packing fraction. MCT shows there is a certain critical packing fraction below which the system exhibit ergodic liquid dynamics and above non-ergodic glass dynamics^23^. If the atomic or molecular interaction in a system is governed by simple potentials, the autocorrelation function of the relevant system becomes stretched in time and the accompanying dynamic transition is a fold bifurcation singularity. If the interatomic or intermolecular potential is complicated, for example in the case of hard-sphere repulsion and short range attraction, the accompanying dynamics become entirely different and higher order glass-transition like cusp and swallow-tail singularities have been observed at the critical packing fraction^24^. The autocorrelation function of these systems is found to decay logarithmically with time close to the higher-order singularities. In recent years, the logarithmic decay of autocorrelation function has been observed in a wide variety of complex systems such as spin glass^25^, granular materials^26^, colloidal solutions^19^,^27^, polymers^28^ and proteins^29^,^30^. Similarly, molecular dynamics simulations have shown logarithmic relaxation of the autocorrelation function to be observed for a wide variety of samples like short-range attractive colloids^31^, polymer blend^32^, protein folding^33^, and kinetically constrained models^34^. However, the influence hydrophobic and hydrophilic nano-confinements on the logarithmic relaxation process is yet to be uncovered. In this letter, we have studied the influence of hydrophobic and hydrophilic nano-confinements on the logarithmic relaxation process and its effect on the MCT critical temperature of salol using quasi-elastic neuron scattering (QENS).

## Results

**Figure 1:**
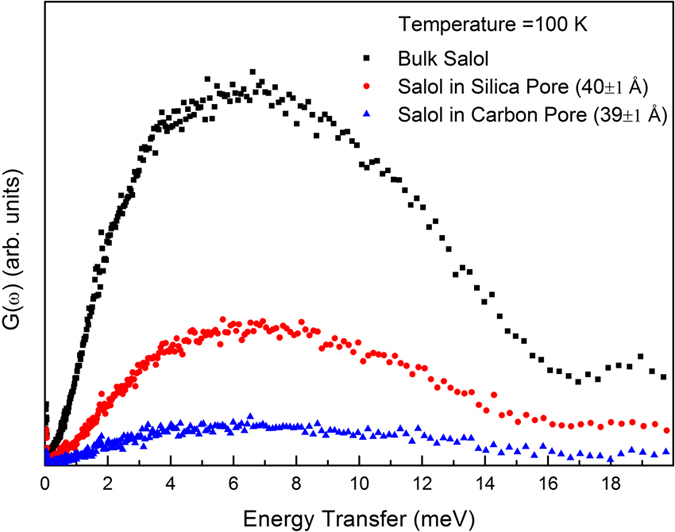
The vibrational density-of-states for bulk salol and salol confined in Carbon pores (39 ± 1 Å) and Silica pores (40 ± 1 Å) at 100 K.

The dynamic structure factor, S(Q, ω) was obtained for bulk and confined Salol at 100 K. The data were corrected for background, multiple scattering and multi-phonon corrections. These data were then summed over all the accessible Q range for the current experimental setup and normalised to the mass of Salol in the beam. The generalized density-of-states G(ω) for each sample was then calculated using the standard expression:


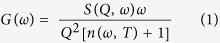


where 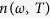 is the Bose population factor^35^. The resulting vibrational density-of-states for bulk salol, salol confined in carbon pores (39 ± 1 Å) and silica pores (40 ± 1 Å) are shown in Fig. 1. The peak around 6 meV is associated with the translative vibrational motions (Boson peak)^36^. As compared to the bulk sample the translative vibrational motions of confined samples are much damped which is visible from the Fig. 1, indicating the reduction of this degree of freedom upon confinement and slowing down of translative vibrational motion. The suppression of this low energy translative vibrational motion of confined salol molecules must be due to strong hydrogen bond interactions between salol molecules and the surface of the nonporous matrix materials. In addition, the translational peak is less affected for salol in silica pores than that in carbon pores. This indicates that the porous silica material provides a much more hydrophobic environment for salol compared with carbon pores.

**Figure 2:**
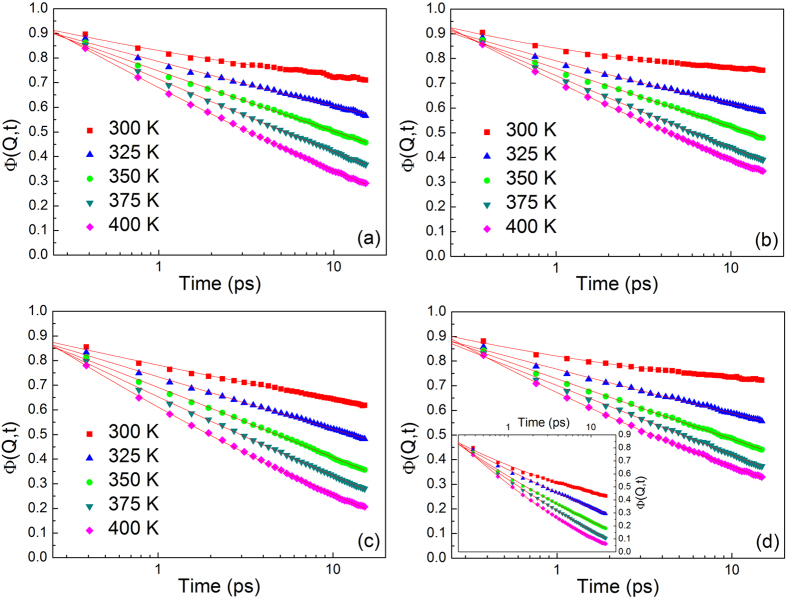
Self-intermediate scattering function of salol in different states at different measured temperatures with a Q value of 1.0 Å^−1^. Salol confined in Carbon pore (39 ± 1 Å) (**a**), Carbon pore (56 ± 1 Å) (**b**), Silica pore (40 ± 1 Å) (**c**) and Silica pore (60 ± 1 Å) (**d**). Inset: the self-intermediate scattering function of bulk salol. The solid lines are fits with equation (3).

The self-intermediate scattering function, Φ(*Q, t*) of salol in the bulk as well as in the confined states were obtained by Fourier transformation of S(*Q, ω*), deconvolution of the instrument resolution, and normalization with the value at t = 0. The bulk salol shows a clear logarithmic relaxation process on the timescale of 0.2–15 ps while confinement of salol in either carbon or silica pores did not hinder the logarithmic relaxation process (see Fig. 2). The following asymptotic formula derived from mode-coupling theory (MCT) is used for expressing the self-intermediate scattering function^37^,^38^,^39^.


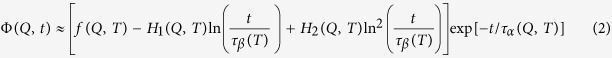


where *f*(*Q, T*) is the Debye−Waller factor i.e., 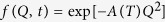, *τ*_*α*_(*Q, T*) and *τ*_*β*_(*T*) are the characteristic *α*-relaxation and *β*-relaxation times, respectively and *τ*_*β*_(*T*) is Q independent. *H*_*1*_(*Q, T*) and *H*_*2*_(*Q, T*) can be written as 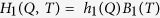 and 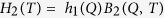, respectively. The *H*_*1*_(*Q, T*) and *H*_*2*_(*Q, T*) represent the first- and second-order logarithmic decay parameters, respectively.

The time range (0.1 ps to 15 ps) of our experiment is much shorter than the *α*-relaxation range^40^, in this case the last exponential factor in equation (2) is close to unity, and we can simplify the asymptotic expression for fitting Φ (*Q, t*) to:


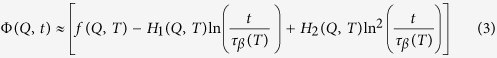


**Figure 3:**
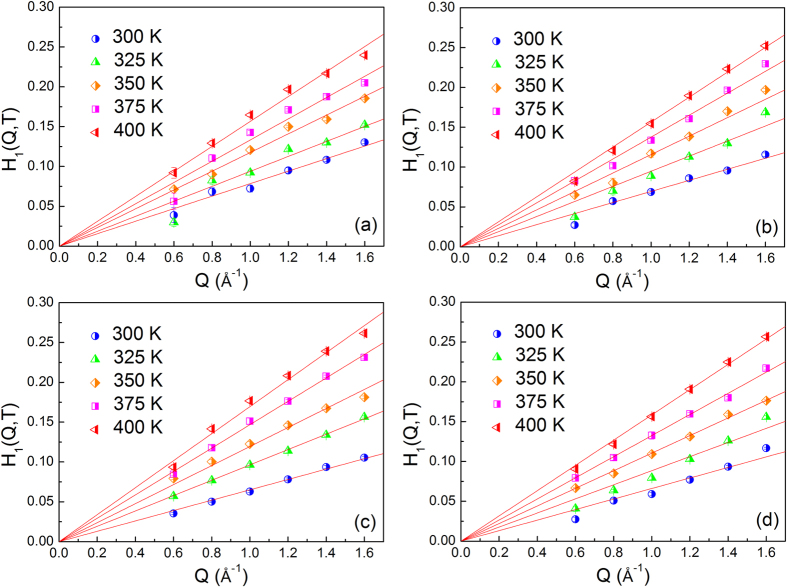
Fitting parameter H_1_(Q, T) of salol in different states as a function of Q at different temperatures. Salol confined in Carbon pore (39 ± 1 Å) (**a**), Carbon pore (56 ± 1 Å) (**b**), Silica pore (40 ± 1 Å) (**c**) and Silica pore (60 ± 1 Å) (**d**).

**Figure 4:**
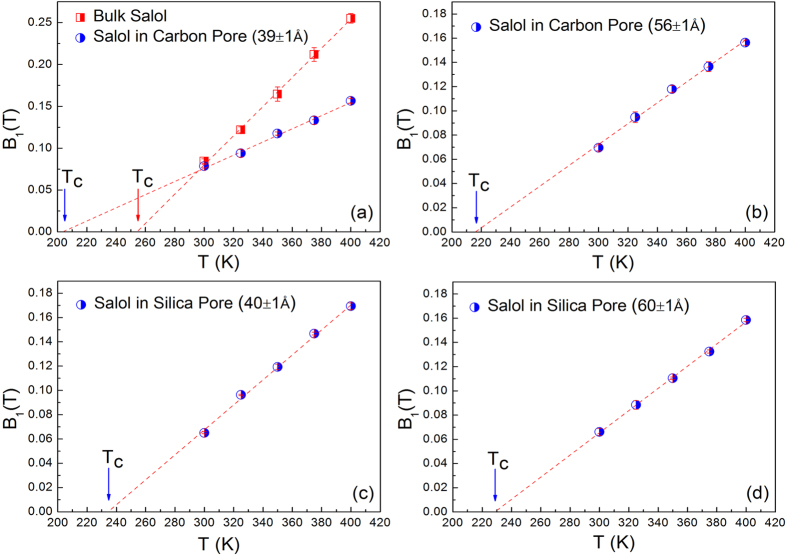
The temperature dependence of B_1_(*T*) for salol in different states. Bulk salol and salol confined in Carbon pore (39 ± 1 Å) (**a**), Carbon pore (56 ± 1 Å) (**b**), Silica pore (40 ± 1 Å) (**c**) and Silica pore (60 ± 1 Å) (**d**). The linear fitting lines are extrapolated consistently to get the mode-coupling crossover temperature, T_c_.

The parameters *f*(*Q, T*), *H*_*1*_(*Q, T*), *H*_*2*_(*Q, T*) and *τ*_*β*_(*T*) are obtained by fitting the curves in the measured time range. The Q dependence of the parameter *H*_*1*_(*Q, T*) is shown in Fig. 3. The solid line is the fitting line by *H*_*1*_(*Q, T*) = B_1_(*T*)*Q*^*b*^, where *b* can take a value between 1 and 2 for small Qs. The fitted B_1_(*T*) values are plotted as a function of temperature in Fig. 4. The Q and temperature dependence of other parameters are provided in the supplementary information (Figures 1S, 2S, and 3S). The temperature dependence of *B*_*1*_(*T*) is extrapolated consistently to get the MCT crossover temperature, *T*_*c*_, also known as MCT critical temperature. According to MCT at this temperature long range liquid like molecular motion freezes and the transport mechanism becomes more like that of the hopping process in a solid^41^. The *T*_*c*_ of bulk salol is 255 ± 2 K, in reasonable agreement with the previous findings^40^,^42^. As expected, this value is about 40 K above the calorimetric glass-transition temperature *T*_*g*_ (218 K), it is universally observed that the *T*_*c*_ is approximately 1.2*T*_*g*_^43^. The present QENS data of salol confined in 39 ± 1 Å and 56 ± 1 Å carbon pores show the *T*_*c*_ are 204 ± 2 K and 214 ± 2 K, respectively. As the pore size decreases *T*_*c*_ also decreases and this observation is similar to that of other reported results^1^. However, in the silica pores with similar pore sizes the freezing temperature did not show an appreciable change. The freezing temperature obtained from the present QENS data of salol confined in 40 ± 1 Å and 60 ± 1 Å silica pores are 234 ± 2 K and 229 ± 2 K, respectively. The freezing temperature of salol in the confining geometry is shifted ~50 K and ~20 K to lower temperatures in the carbon pore (39 ± 1 Å) and silica pore (40 ± 1 Å), respectively. These results show that the microscopic glass-transition temperature is about 30 K lower in 39 ± 1 Å hydrophilic carbon pores than 40 ± 1 Å hydrophobic silica pores. This reduction in the critical temperature is unusual, for example, by confining water in a hydrophilic porous silica matrix MCM-41-S (25 Å pore diameter), Faraone *et al*. reported the phenomenon of a dynamic crossover at *T*_*L*_ ≈ 225 K^44^. In contrast, water confined in a hydrophobic substrate exhibits a lower dynamic crossover temperature at *T*_*L*_ ≈ 190 K^13^.

Experiments have shown that all liquids confined in nanopores with relatively weakly attractive pore walls (hydrophobic) exhibit a lowering of freezing temperature than the bulk freezing temperature^2^. The phenomenon of decrease of the freezing point has been observed for cyclohexane and benzene confined in weakly adsorbing porous silica materials^26^. The freezing point depression was found to be larger for cyclohexane than for benzene, this implies that the interaction of benzene with the wall was stronger than that of cyclohexane. In nanopores with strongly attractive pore walls (hydrophilic), generally, liquids freeze at lower temperature than the bulk but the depression of the freezing temperature is not as low as that of hydrophobic confinements. Even increases in the freezing temperature over the bulk value has been reported for liquids confined in strongly attractive pore walls^46^. Differential scanning calorimetry experiments by Kaneko and co-workers show that confining benzene or carbon tetrachloride in activated carbon fibres with pore size in the range of 11 Å–17 Å increases the freezing temperature^46^. It is universal that the freezing point depression of liquids in hydrophobic confinements is larger than hydrophilic confinements. Our results, in contrast, show that, although both hydrophobic and hydrophilic confinements decrease the freezing temperature of salol, the hydrophilic confinements show a larger depression of the freezing temperature. This result is unusual and we believe that this phenomenon is directly linked to the unique in-cage rattling (β-relaxation) process of salol molecules; i.e. the logarithmic relaxation process. All the above mentioned other liquids: water, benzene, cyclohexane and carbon tetrachloride do not exhibit the logarithmic relaxation process rather the β-relaxation process exhibits an exponential decay with time.

In summary, we have presented the first experimental evidence of an unusual lowering of MCT critical temperature of a liquid that shows a logarithmic β-relaxation process in nanoscale confined states. The logarithmic β-relaxation process exhibited by salol in its bulk state is found to exist in the hydrophobic and hydrophilic nanoscale confinements. The generalized vibrational density-of-states of confined salol indicates that the carbon nanopores offered a hydrophilic confinement whereas silica pores presented a hydrophobic confinement for the salol. The freezing temperature of the confined salol is found to be lower than that of the bulk. However, in the hydrophilic confinements the lowering of the critical temperature of the salol found to be much larger than that of the hydrophobic confined state. This observation is in contrast to the general behaviour that in the hydrophobic confinement the critical temperature of liquids is much lower as compared to the hydrophilic confinements. The present experimental results indicate that the molecules that exhibit logarithmic relaxation process undergo a unique transition to glassy state in nanoscale confinements.

## Methods

Salol (Phenyl-salicylate, C_13_H_10_O_3_) or 2-hydroxy benzoic acid phenyl ester is known to exhibit logarithmic decay of the β-relaxation and it is a well-known glass-forming liquid. The *T*_*g*_ and *T*_*m*_ of the bulk salol are 218 K and 315 K, respectively. To confine the salol, we chose ordered nano-porous carbon with pore sizes of 39 ± 1 Å (BET800) and 56 ± 1 Å (BET1000) and nano-porous silica gel with 40 ± 1 Å and 60 ± 1 Å, which were brought from ACS materials and Sigma-Aldrich, respectively. The pore size distribution of the materials have been characterized by the supplier using gas absorption analysis. The salol was loaded in the pores of above materials by an established liquid impregnation method which resulted in the pores being 100% filled^47^. The QENS experiments were done on the PELICAN spectrometer at the Bragg institute, Sydney, Australia^48^. Using the neutron wavelength of 6.0 Å, the accessible Q range was 0.4 Å^−1^ to 1.8 Å^−1^, with a resolution of 75 μeV. Therefore, the experimental setup covers a dynamical time window of 0.1~15 ps. The QENS data were collected at 5 different temperatures, from T = 300 K up to 400 K in 25 K intervals. In addition, the samples were measured at 100 K in order to get the instrumental resolution function. Because of the high incoherent neutron cross-section of hydrogen and the Salol contain 10 hydrogen atoms per molecule the scattered signals mainly due to incoherent scattering process. The data were collected for 3 hours at each temperature. An empty sample container was also measured at each temperature, and scattering from a vanadium sample with similar sample geometry was used to normalize the scattered signals. The dynamic structure factors S(*Q, ω*) of the hydrogen atom in salol samples were obtained by careful subtraction of the background, correcting for self-absorption and container scattering and interpolation to constant wave numbers Q, and multiplying with the detailed balanced factor.

The generalized vibrational density-of-states of salol are derived by the following steps. In inelastic neutron scattering the measured quantity can be described by the neutron scattering cross section per unit solid angle dΩ and per unit energy dE.


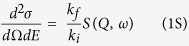


where S(Q, ω) is the dynamic structure factor, Q and ω are neutron momentum and energy transfer. *k*_*i*_ and *k*_*f*_ are neutron wave vector before and after scattering. Since a salol molecule has 10 hydrogen atoms and the incoherent scattering cross section of hydrogen is a factor of 10 more than that of scattering cross section (incoherent or coherent) of any other elements the dynamic structure factor in this equation can be rewritten as


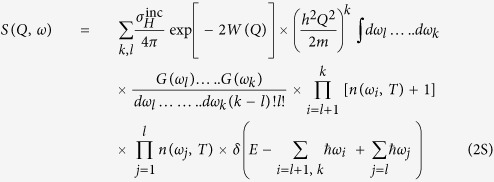


here *n*(*ω, T*) is the Bose population factor *m* is the neutron mass and W(Q) is the Debye-Waller factor of hydrogen atoms and *l* and *k* are creation and annihilation of excitation. G(ω) is the generalized vibrational density-of-states of hydrogen atoms, which can be describe summation of all general modes as


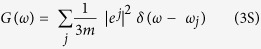


where **e**^**j**^ is the polarization vector of normal modes and *m* is hydrogen atomic mass. The generalized phonon density-of-states plotted in the Fig. 1 are normalized with weighted macroscopic scattering cross section of Salol on the neutron beam. Equation 2S can be used to estimate the multiphonon contributions by an iterative technique. At the first iterative step, *G*(ω) and *W*(*Q*) could be calculated from the experimental data based on an assumption that the neutron scattering spectrum of salol is the one-phonon spectrum. At subsequent steps, the difference between the experimental spectrum and spectrum resulting from the multiphonon processes is accepted as the new one-phonon spectrum. The one-phonon part of dynamical structure factor *S*(*Q*, ω) for INS spectrum


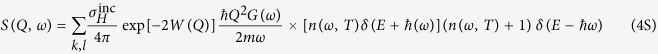


This equation is directly related to generalized vibrational density of states *G*(ω) as


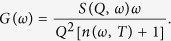


## Additional Information

**How to cite this article**: Chen, C. *et al*. The logarithmic relaxation process and the critical temperature of liquids in nano-confined states. *Sci. Rep.* **6**, 33374; doi: 10.1038/srep33374 (2016).

## Supplementary Material

Supplementary Information
