# Supplementary material for: The logarithmic relaxation process and the critical temperature of liquids in nano-confined states
Source: Sci Rep. 2016 Sep 27;6:33374. doi: 10.1038/srep33374 (PMC5037365; doi:10.1038/srep33374)
Supplement: Supplementary Information [file srep33374-s1.pdf]

Supplementary Information For

The logarithmic relaxation process and freezing of liquids in the nano-confined states

Changjiu Chen[1, 2], Kaikin Wong[1,2], Richard A. Mole[3], Dehong Yu[3] and Suresh M. Chathoth[1,2*]

[1] Department of Physics and Materials Science, City University of Hong Kong, Hong Kong, 999077, P. R. China

[2] City University of Hong Kong Shenzhen Research Institute, Shenzhen, 518057, P. R. China

[3] The Bragg Institute, Australian Nuclear Science and Technology Organization, Lucas Height, 2234, Australia

*Email: (SMC) smavilac@cityu.edu.hk

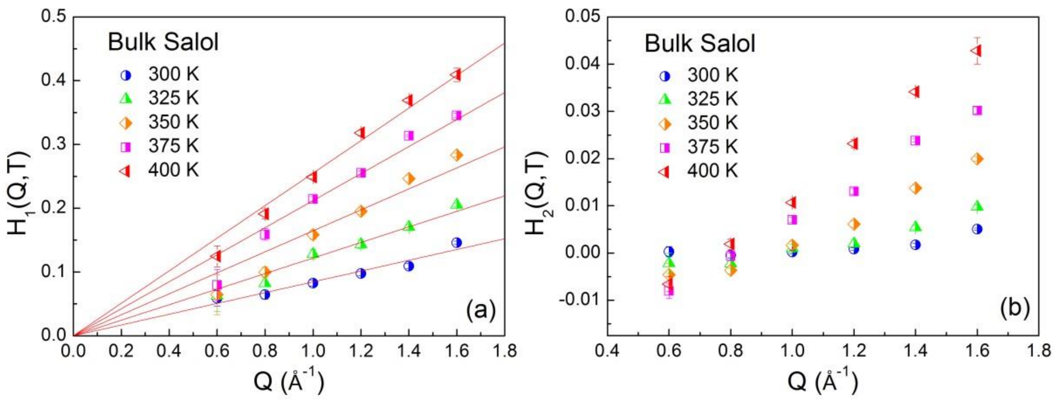

Figure 1S. Fitting parameter $H_1(Q,T)$ (a) and $H_2(Q,T)$ (b) of bulk salol as a function of Q at different temperatures.

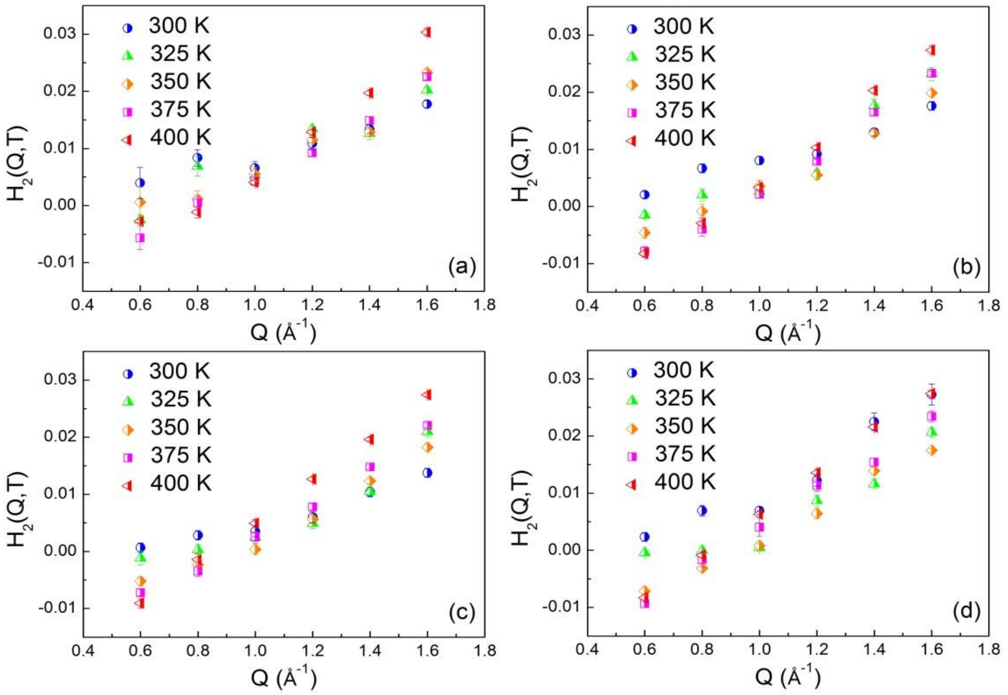

Figure 2S. Fitting parameter $H_2(Q,T)$ of salol in different states as a function of Q at different temperatures. Salol confined in Carbon pore (39±1 Å) (a), Carbon pore (56±1 Å) (b), Silica pore (40±1 Å) (c) and Silica pore (60±1 Å) (d).

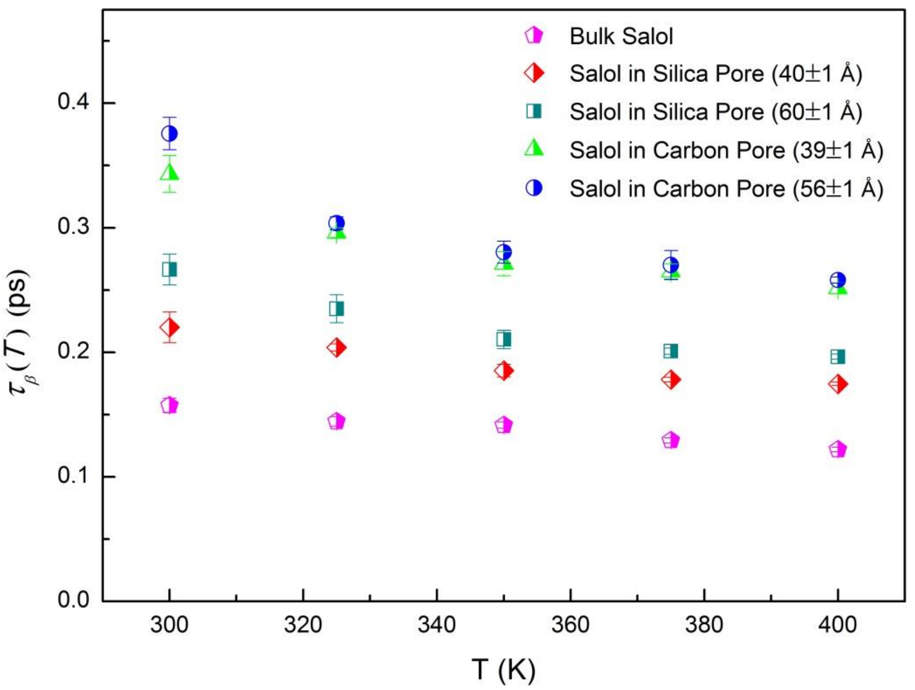

Figure 3S. The temperature dependence of $\tau_\beta(T)$ for bulk salol and salol in different confinements.
